# Effects of a sensory-motor orthotic on postural instability rehabilitation in Parkinson’s disease: a pilot study

**DOI:** 10.1186/s40734-017-0058-y

**Published:** 2017-07-06

**Authors:** Daniele Volpe, Elisa Pelosin, Leila Bakdounes, Stefano Masiero, Giannettore Bertagnoni, Chiara Sorbera, Maria Giulia Giantin

**Affiliations:** 1 Department of Physical Medicine and Rehabilitation, Neurorehabilitation Unit “Villa Margherita,”, Via Costacolonna n.6 Arcugnano, Vicenza, Italy; 2 Department of Neuroscience, University of Genoa, Genoa, Italy; 3 School of Physical Medicine and Rehabilitation, University of Padua, Padua, Italy; 4 Department of Physical Medicine and Rehabilitation, S. Bortolo Hospital, Vicenza, Italy

**Keywords:** Parkinson’s disease, Sensory-motor orthotic, Postural instability, Rehabilitation

## Abstract

**Background:**

Proprioceptive deficits have been largely documented in PD patients, thus external sensory signals (peripheral sensory feedback) are often used to compensate the abnormalities of proprioceptive integration.

This pilot study aims to evaluate the feasibility and the effectiveness of a rehabilitation-training program, combined with the use of a sensory-motor orthotic in improving balance in a small sample of PD patients.

**Methods:**

Twenty PD patients were randomly allocated into two groups: (i) *the Experimental group*, where participants were asked to wear a sensory-motor orthotic during the balance training program and (ii) *the Control group*, where subjects performed an identical training program without wearing any kind of orthotics. In all, the training program lasted 10 sessions (5 days a week for 2 weeks) and the clinical and instrumental assessments were performed at baseline, immediately after the end of the training and 4 weeks after the rehabilitative program was stopped.

**Results:**

All clinical outcome measures tested improved significantly at post and follow-up evaluations in both groups. Interestingly, at the end of the training, only the experimental group obtained a significant improvement in the functional reaching test (sway area - eyes closed) measured by means of stabilometric platform and this result was maintained in the follow-up evaluation.

**Conclusions:**

Our preliminary results suggested that the use of a sensory-motor orthotic, in combination with a tailored balance training, is feasible and it seems to positively impact on balance performance in Parkinson’s disease.

**Trial registration:**

EudraCT N. 003020–36 - 2013.

## Background

Parkinson’s disease (PD) is a neurological progressive disorder characterized by balance dysfunctions, often associated with the high risk of falling [1] that negatively impacts on the quality of life [2]. In PD, most of the falls occur during a sudden change of posture or during walking [3] in various circumstances (i.e., gait initiation, dual task conditions). Balance problems, in PD patients, are probably due to the overlapping of different factors, such as stopped posture, deficits in postural responses [4], reduced limit of stability [5] and impaired executive function (i.e., deficit in selective attention) [6]. Although much is known about the multifactorial nature of gait disturbances and falls in PD, the pathophysiology of postural instability is still unclear. It seems to depend on a complex interactions between the impairment caused by the disease at different levels of the nervous system and compensatory strategies [7, 8]. It is well- known that postural control in PD patients mainly relies on visual information, which is possibly used for compensating proprioceptive impairments [9, 10]. Indeed, PD patients seem to have somatosensory abnormalities with abnormal proprioceptive (kinesthetic) processing that produces a reduced perception of passive motion limb position [11, 12] and space orientation [13]. Therefore, abnormalities in sensory processing have been suggested to play a major role in the pathogenesis of sensory dysfunctions in PD [14]. Some authors demonstrated that in a gravity environment, healthy subjects mainly rely on somatosensory information in order to maintain an upright posture [15] and that artificially impairing proprioception worsens postural stability, particularly reducing the COP displacements in response to external perturbations during visual deprivation [16]. In fact, in PD, a defective scaling and habituation of postural reactions during either neck or leg vibration has been revealed [17, 18].

Beside the poor effect of dopaminergic treatment in improving balance problems, the effects of physical activity and exercise programs on improving balance [19–21] and quality of life [21] have been extensively proven in patients with PD. However, the possibility of enhancing training effects, by combining intervention with proprioceptive orthotic, has never been tested.

Proprioceptive rehabilitation aims to improve or enhance the perception of proprioceptive signals and their central integration, thus possibly compensating the impaired “gating” function of the basal ganglia [22]. Furthermore, external sensory signals (peripheral sensory feedback) can be used to compensate the abnormal sensorimotor integration in PD patients [23]. Moreover, muscle spindle endings respond to proprioceptive stimulations with an increased muscular activation, thus producing a tonic contraction on the stimulated muscle [24, 25].

**Fig. 1:**
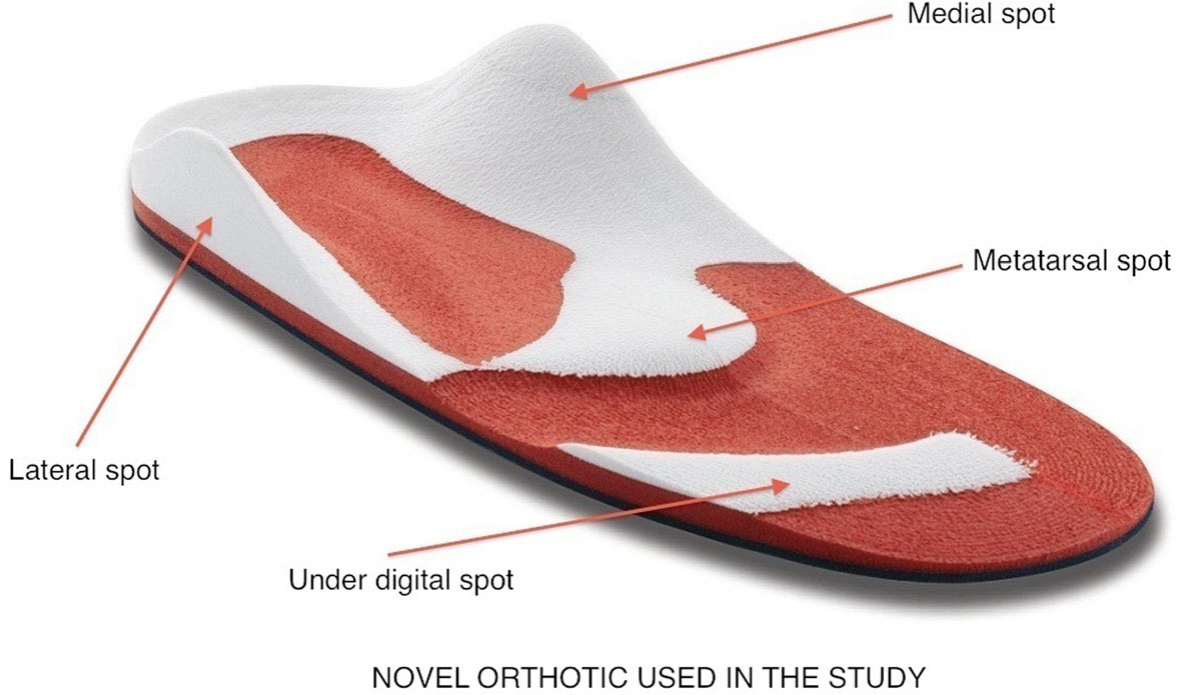
Example of the sensory-motor orthotic

In detail, the sensory-motor (SM) orthotic [Fig. 1] used in this study, combines biomechanical and sensory-motor input on the plantar surface of the feet by modulating through function activation of specific muscle groups. In fact, it has been demonstrated that tendon stimulation has an influence on muscular tone with increased voluntary activation and improved muscle velocity and strength [26, 27]. The proposed novel orthotic is composed of four spots, which through muscle tendon stimulation exerts a compression which activates anticipated muscle contractions: a) the medial spot which activates the medial muscular kinetic chain (tibia, adductor, paraspinal muscles); b) the lateral spot which activates the lateral muscular kinetic chain (peroneal, abductor, iliotibial, paraspinal muscles muscles); c) the metatarsal and under digital spots which stimulate the extensor muscular kinetic chain (fingers flexors, triceps, femoris biceps s, gluteus and paraspinal muscles). No prior study of SM orthosis on balance dysfunctions in PD has been published before. We have no evidence to support this hypothetical mechanism of function.

The present study aims (i) to explore the feasibility and the safety of using a Sensory-Motor orthotic as a tool of increasing plantar proprioceptive information and (ii) investigating if the combination of the SM orthotic, with a balance training, might enhance postural control, balance and gait in a small group of PD patients.

## Methods

### Participants

A total of 30 patients with idiopathic PD, according to the United Kingdom Parkinson’s Disease Society Brain Bank criteria [28], were recruited from the Department of Neurorehabilitation in Villa Margherita, Arcugnano (Vicenza), Italy.

Participants were enrolled in the study if they met the following inclusion criteria: stage 3 of the Hoehn and Yahr (H&Y) scale, Mini Mental State Examination (MMSE) [29] with score > 24, ability to walk independently without a walking aid and to attend a physiotherapy venue, the absence of serious co-morbidities (cardiac, pulmonary or orthopaedic diseases) that could impact gait or balance. Patients were excluded if they suffered from major depression (diagnosed by means of a Diagnostic and Statistical Manual of Mental Disorders - DSM V criteria), had Deep Brain Stimulation implants, were medically unstable or had medication induced (dyskinesias), had an history of other conditions affecting stability (e.g., poor visual acuity or vestibular dysfunction, neuropathy or sensory ataxia). In this pilot study, we recruited patients in stage 3 of H&Y scale exclusively. Thus, all patients were in a moderate stage of PD and had balance problems probably due to abnormal sensory motor integration. In addition, as this was a pilot study, we selected only PD in H&Y = 3 because we wanted to limit, as much as possible, the heterogeneity amongst the patients recruited. At the end of the screening phase, twenty patients with PD were enrolled in the study and ten patients were excluded because six participants did not meet the inclusion criteria (*n* = 1 had MMSE > 24; *n* = 2 needed assistance during walking; *n* = 2 had DBS and *n* = 1 had severe dyskinesia) and four patients were unable to attend the physiotherapy program due to personal reasons.

### Study design (Fig. 2)

**Fig. 2:**
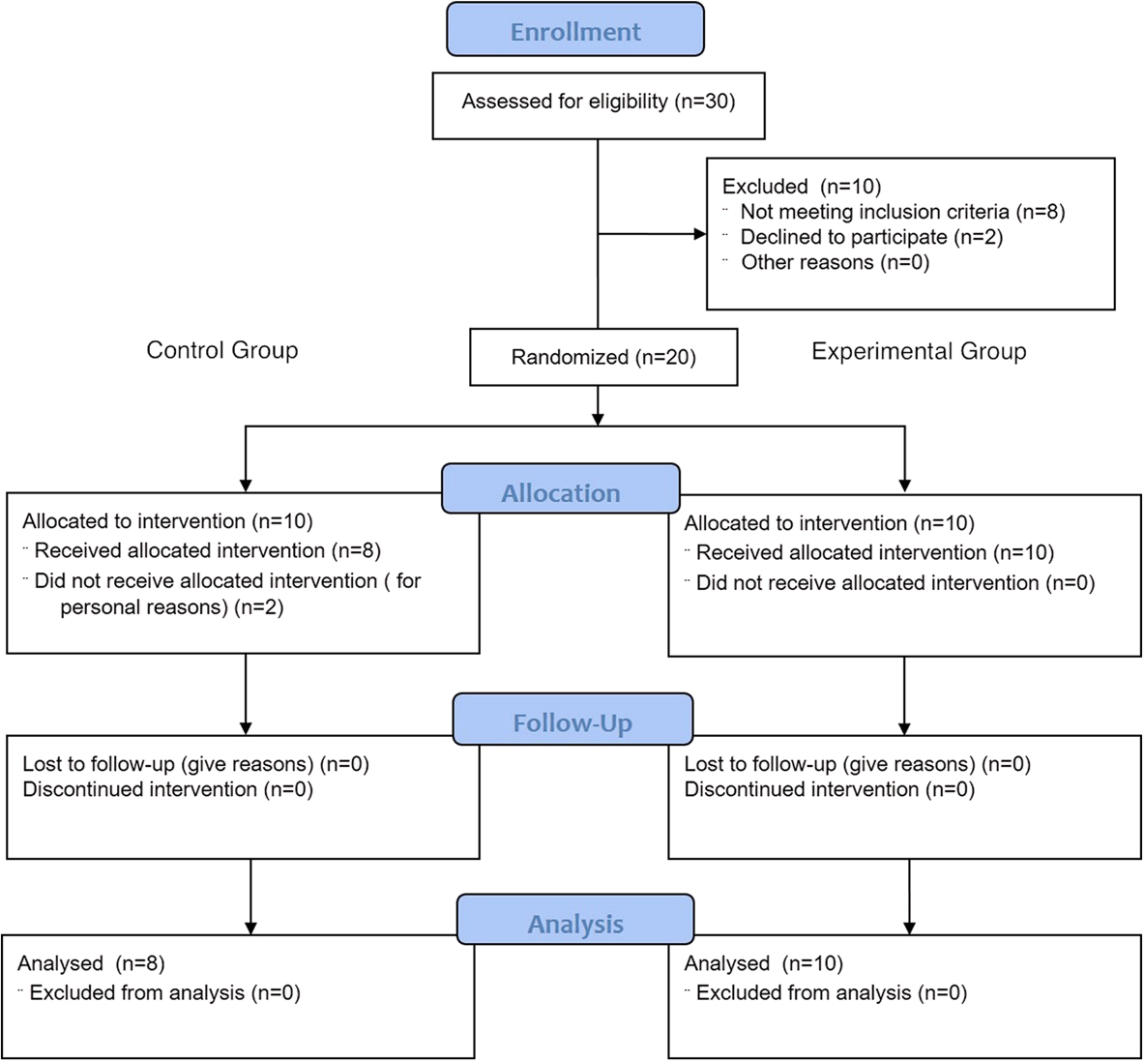
CONSORT 2010 Flow Diagram. Effects of a Sensori-Motor Orthotic on Rehabilitation of Postural Instability in Parkinson’s disease: a Phase II Randomized Pilot Study

In this pilot study, after the initial screening procedures, participants were randomly allocated into two groups: (i) *The Experimental group*, in which participants were asked to wear a SM orthotic before and after the training program or (ii) *The Control group*, where subjects performed an identical training program without wearing any kind of orthotics.

All the clinical and instrumental assessments were performed at baseline (PRE - within 1 week before the beginning of the intervention), after the end of the training (POST - within two days after the last training session) and 4 weeks after the completion of the rehabilitative program (FU - follow-up assessment). Randomization procedure, conducted by a third party, was used to allocate participants to one of the two treatment groups (i.e., experimental or control groups). The assessors were blinded to the group allocation during the whole duration of the study. The study coordinator responsible for the SM orthotics supervision was not blinded to the group allocation, but he was not involved in rehabilitation procedures or outcome assessments. The physiotherapists providing the training program were blinded and not involved in other aspects of the trial (i.e., aims, hypothesis or predictions of the study were not disclosed).

### Interventions

All PD subjects underwent a training balance program composed by 10 sessions (5 days a week for 2 weeks). Each session lasted 50 min and the exercises were identical for both groups. Table 1 details the type of daily balance training program provided by the hospital physiotherapists in accordance to the Koninklijk Nederlands Genootschap voor Fysiotherapie - KNGF Guidelines for Physiotherapy. At the beginning of each session, participants were required to sign a form in order to attest their attendance. The physiotherapy protocol included 30 min of exercises designed to improve balance. Precisely, intervention included a perturbation-based balance-training program, where patients were asked to voluntarily reach their limit of stability. During these exercises, participants were required to concentrate and activate the appropriate postural responses in order to react to the external perturbations. Balance training was preceded by warming up and stretching exercises and followed by a cooling down period. Each phase lasted approximately 10 min. Subjects who enrolled in the Experimental group were required for the entire duration of the study (2 weeks) to wear the SM orthotics all day long except during the training balance sessions.

**Table 1:** KNGF Guidelines: physiotherapy program for balance training

Improvement of physical capacity	To maintain or to improve physical capacity with training of aerobic muscle strength (with the emphasis on the muscles of the trunk and legs), joint mobility (among others, axial) and muscle length (among others, muscles of the calf and the hamstrings, flexor and extensor of the knee)
Improvement of the transfers	To train transfers by applying cognitive improvement strategies and cues to initiate and continue movements
Normalizing body posture	To prevent or treat postural deformities with exercises for postural alignment and coordinated movements
Training balance	To optimize balance during the performance of activities in static and dynamic conditions with exercises for training strength and perturbation-based balance training with emphasis of functional reaching test in protected condition and how to activate postural responses to perturbation. Falls prevention strategies.
Gait training	To walk safely and to increase (comfortable) walking speed with exercise walking with the use of cues and cognitive movement strategies and to train muscle strength and mobility of the trunk and upper and lower limbs.

### Clinical and instrumental tests

#### Clinical assessments

Motor impairment was assessed during the III section (motor examination) of the Unified Parkinson’s disease Rating Scale (UPDRS) [30], the Berg Balance Scale (BBS) [31], the Timed Up and Go (TUG) [32], and the Six-minute Walking Test (6mWT) [33]. We also quantified the health-related quality of life in all participants using the Parkinson’s Disease Questionnaire (PDQ-39) [34]. All adverse events such as injuries, distress and hospital admissions were verified by phone interviews and recordings taken during the pilot study period.

#### Posturography assessments

Static posturography was assessed in keeping with current guidelines [35]. The Center of Pressure (CoP) excursion was recorded by means of a force platform (Milletrix model 2.0–Rome, Italy). All data were collected with a 50 Hz sampling frequency. The CoP was recorded during an upright stance in a quiet room. Participants were instructed to stand erect, with their arms alongside their body. Their feet were kept at an angle of about 30° opened to the front and with the heels approximately 3 cm apart. Furthermore, an instrumental version of the functional reaching test (FRT) [36] was executed by asking the subject to elevate their arm to shoulder’s height and then to perform a maximum forward reach, while maintaining the heel on the platform with their feet planted in a standing position.

In all the tasks, data was collected for 51.2 s, in both eyes opened (EO) and eyes closed (EC) conditions. The following parameters were taken into account: the sway area (mm^2^), measured as the 95th percentile of an ellipse fitted to the overall CoP trace; COP velocity (mm/s) and the Romberg index. These parameters were chosen as a tool to evaluate CoM displacement during sway as a response to perturbation.

### Statistical analysis

Demographic and clinical characteristics between the two intervention groups of PD (Experimental and Control) were tested by means of Chi-square (*χ* ^2^) test (gender) and *t*-test (age, UPDRS - Part III Motor, and disease duration) statistics. All clinical and instrumental variables were examined for normality (Shapiro-Wilk W test), and mean and standard deviation (SD) were calculated. For the analysis a Repeated Measures (RM) Analysis of Variance (ANOVA) was used with Group (Experimental, Control) as between-subjects factor and Time (Baseline, Post and Follow-up) as within-subjects factor. The pre-defined level of significance was set at *p* < 0.05. Post hoc analysis of significant interactions was performed by means of -tests applying the Bonferroni correction for multiple comparisons when necessary. All statistical analyses were performed with SPSS 22.0.

## Results

At the end of the study, two patients were excluded from the analysis because they dropped out from the training protocol due to personal reasons. Patients with PD enrolled into two groups, did not differ for demographic, clinical characteristics (Table 2) and clinical assessment (p always > 0.05) recorded at the baseline. For the sample as a whole, 100% of intervention sessions were delivered across study arms. No major adverse event or death was recorded during the study period.

**Table 2:** Baseline demographic and clinical variables of the two groups enrolled in the study

	EXP Group	CTRL Group	Statistics
	mean ± SD	mean ± SD	Baseline
Gender (M/F)	7/3	5/3	
Age (yr)	69.18 ± 7.61	63.37 ± 6.89	*p* = 0.24
Height (cm)	160.91 ± 9.58	160.62 ± 14.74	*p* = 0.96
Weight (kg)	69.54 ± 13.33	67.62 ± 8.31	*p* = 0.72
Disease duration (yr)	7.82 ± 4.00	8.12 ± 2.90	*p* = 0.86
Falls (n)	1.45 ± 2.16	0.87 ± 0.99	*p* = 0.07
Levodopa (mg/day)	455.32 ± 355.49	409.19 ± 340.68	*p* = 0.74
• *Dopamine agonist (LEDD mg)*			
Pramipexole E.R.	*n* = 2	*n* = 3	N.A.
Ropirinole E.R.	*n* = 3	*n* = 3	N.A.
Rotigotine (*n* = 1)	*n* = 1	*n* = 1	N.A.
Rasagiline (*n* = 1)	*n* = 2	*n* = 1	N.A.
• *Other drugs (LEDD mg)*			
Entacapone	*n* = 1	*n* = 2	N.A.
Selegiline	*n* = 1	*n* = 2	N.A.
Amantadine	*n* = 2	*n* = 2	N.A.

Exp, Experimental; CTRL, Control; M, Male; F, Female; Yr, Years; Cm, centimeters; Kg, Kilograms; Mg = Milligrams; N, number; ER = Extended Released; N.A., Not Applicable

### Clinical assessments

All data [mean ± standard deviation (SD)] collected at baseline, post and follow-up examinations are reported in Table 3. Statistical analysis showed a positive effect of the balance-training program with no differences between groups in all the variables considered. Precisely, the mean score of UPDRS-III was significantly reduced in the Experimental as well as in the Control groups. RM-ANOVA revealed a significant effect of TIME (*p* < 0.01), without any significant Time X Group interaction (*p* = 0.41). Interestingly, improvements were seen both immediately after the training and at the FU examination (p always < 0.01).

**Table 3:** Clinical variables of the two groups enrolled in the study and their comparisons at each time point

	PSM Group	CTRL Group	Statistic post-hoc TIME
Motor UPDRS section III at T0-Baseline	40.87 ± 6.01	39.00 ± 11.89	
Motor UPDRS section III at T1-Discharge	37.12 ± 6.66	36.90 ± 12.02	*p* < 0.01
Motor UPDRS section III at T2-Follow up	35.55 ± 6.57	36.80 ± 11.80	*p* < 0.01
Berg Balance Scale T0-Baseline	45.63 ± 5.92	45.12 ± 4.58	
Berg Balance Scale T1-Discharge	49.3 ± 3.15	47.12 ± 5.05	*p* < 0.01
Berg Balance Scale T2-Follow up	50.1 ± 2.72	49.37 ± 5.35	*p* < 0.01
Falls T0-Baseline	1.45 ± 2.16	0.87 ± 0.99	
Falls T1-Discharge	0.45 ± 1.03	0.12 ± 0.31	*p* < 0.01
Falls T2-Follow up	0.00 ± 0.00	0.00 ± 0.00	N.A.
Timed Up and Go T0-Baseline	13.08 ± 2.17	13.8 ± 3.43	
Timed Up and Go T1-Discharge	12.13 ± 1.35	12.8 ± 2.81	*p* = 0.01
Timed Up and Go T2-Follow up	10.81 ± 1.07	13.2 ± 2.75	*p* < 0.01
6MWT T0-Baseline	305.64 ± 48.89	319.8 ± 48.59	
6MWT T1-Discharge	335.64 ± 44.09	332.5 ± 66.00	*p* = 0.03
6MWT T2-Follow up	342.2 ± 59.99	328.38 ± 70.18	*p* = 0.01
PDQ-39 T0-Baseline	57.7 ± 22.93	59 ± 14.38	
PDQ-39 T1-Discharge	54.36 ± 24.47	49.5 ± 20.52	*p* = 0.03
PDQ-39 T2-Follow up	52.1 ± 27.44	51.25 ± 19.46	*p* = 0.02

Exp, Experimental, *CTRL* Control, *UPDRS* Unified Parkinson Disease Rating Scale, *6MWT* Six Meters Walking Test, *PDQ-39* Parkinson’s Disease Questionnaire-39 items. N.A., not applicable

*P* values represent the post hoc analysis (T0 vs T1 and T0 vs T2) when a main effect of TIME was detected with Repeated Measures ANOVA

For the tests assessing dynamic balance performance (BBS and TUG), RM-ANOVA showed a main effect of TIME (BBS: *p* < 0.01 and TUG: *p* < 0.01) with no Time X Group interaction. In details, for BBS a significant increase of the total score was seen at Post (*p* < 0.01) and at the FU (*p* < 0.01) evaluations as well as for TUG, where a significant decrease of time in performing the test was seen immediately after the training (*p* = 0.01) and 1 month later (FU: *p* < 0.01). No Time X Group interaction was revealed by the statistical analysis. Similar results were also found in gait resistance performance. Indeed, the analysis of 6MWT data showed a significant effect of Time (*p* = 0.02) with no differences between the two groups. Thus, an overall improvement was seen immediately after the training (Post: *p* = 0.03) and it was maintained at the FU examination (*p* = 0.01). Balance and gait improvements were also confirmed by a significant decrease of fall rate. Indeed, RM-ANOVA showed a main effect of Time (*p* < 0.01) with an improvement at post (*p* = 0.01). However, no significant Time X Interaction was recorded by the statistical analysis (*p* = 0.55). Finally, positive changes on participants’ QoL recorded by means of PDQ-39 questionnaire were seen at the end of the training (Post: *p* = 0.03) as well as the following testing time (FU: *p* = 0.02). Indeed, RM-ANOVA revealed a significant effect of TIME (*p* = 0.02) with no significant Time X Group interaction.

### Posturography

**Fig. 3:**
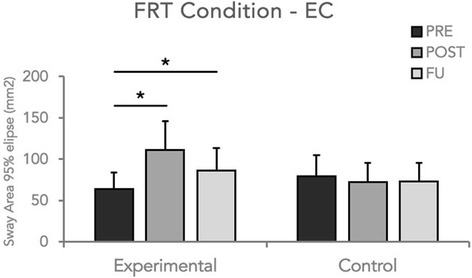
Sway area values during instrumental FRT-EC condition of the two groups enrolled in the study at each time point

**Fig. 4:**
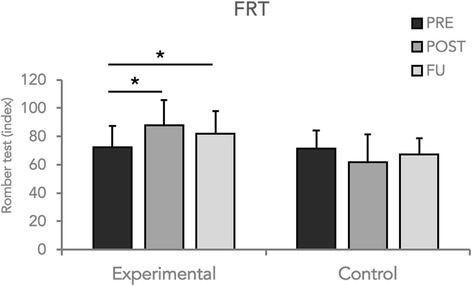
Romberg index values during FRT condition of the two groups enrolled in the study at each time point

Statistical analysis did not reveal significant changes for sway area recorded in the quiet stance test (p always >0.05) in both conditions (EC and EO). However, RM ANOVA showed a significant main effect of Group (*p* = 0.04) and a significant Group x Time interaction (*p* = 0.03) for 95% confidence ellipse area data obtained during the FRT test in the EC condition. Furthermore, post-hoc analysis revealed that only the experimental group obtained a significant improvement at the end of the training period (*p* = 0.02) and this result was maintained at the follow-up examination (Fig. 3). Similar results were also found for the values obtained for the Romberg index. Indeed, statistical analysis (RM-ANOVA) revealed a significance of the factor Group (*p* = 0.04) as well as a significant Group x Time interaction. Post-hoc analysis showed that only in the experimental group, velocity increased at the end of the training (*p* = 0.03) and at the follow-up evaluation (*p* = 0.04) (Fig. 4). No significant changes were detected during static and dynamic (FRT) evaluation under EO condition. Finally, no significant changes were found for CoP velocity in any experimental condition (EC and EO).

## Discussion

The aim of the present study was to explore the feasibility and the safety of using a Sensory-Motor orthotic as a tool for increasing plantar proprioceptive information. Furthermore, it was carried out to verify if the combination of the SM orthotic, with a rehabilitative intervention, could enhance postural control, balance and gait in a group of subjects with PD.

The rehabilitative program was delivered successfully, with a good level of adherence rate confirmed by the patient’s participation and involvement. On the whole, our results demonstrated that combining balance training with a sensory-motor orthotics in a rehabilitation setting is feasible and might lead to some clinically meaningful effect in PD patients with postural instability. However, only subjects enrolled in the experimental protocol significantly improved their limit of stability measured by a stabilometric platform. Precisely, an increase of sway area values, obtained during the instrumental functional reaching test, and an improvement of the Romberg index were seen only in the experimental group immediately after the training and follow-up evaluation. As stated in the introduction, PD-related abnormality in proprioception might manifest itself as alteration of kinesthesia (for a review see [13]. Indeed, PD patients have an impaired sense of the timing [37] and discrimination [38] of proprioceptive inputs, which can also lead to deficient compensation of mechanical perturbations, especially during the activation of anticipatory postural adjustments [39]. The enhancement of the proprioceptive inflow, as that induced by the sensory-motor orthotic used in this study, might overcome the subtle impairment in kinesthesia, as previously argued [37]. PD patients used to have a reduced limit of stability particularly during dynamic conditions, thus pointing to dynamic posturography as a better instrument of capturing improvements in balance [5, 35]. It is well-known that anticipatory postural adjustments and reactive postural reactions in PD are compromised, in the sense that they are reduced in amplitude and velocity [39]. So another possible mechanism of action could be related to the influence on muscles of proprioceptive stimulation exerted by the SM orthotic, since tendon stimulation [40, 41] seems to increase muscular tone and velocity promoting the activation of anticipatory postural adjustments and reactive postural reactions. Finally, it is important to notice that significant changes in the posturographic data during the FRT in the experimental group were seen only when patients were required to execute the test with their eyes closed, a set-up relying on proprioceptive information. This fact might suggest an improvement of proprioceptive signals derived from the effect of the SM orthotic.

This pilot study has a number of limitations. Firstly, even if testing occurred at the peak dose of the morning medications, we cannot rule out the bias introduced by fluctuations in levodopa plasmatic concentration. Secondly, even though the sample size allowed the detection of significant changes, here we reported results obtained in a small group of patients, thus our results have to be replicated by larger trials. Thirdly, due to the shortness of training and the follow-up examination, we did not evaluate changes in fall rates. Further study should have to include episode supervision of falls. Fourthly, even if the physiotherapy program for balance training was conducted in accordance with published guidelines, the execution of exercises were influenced by therapists expertise and patients’ motivation, meaning that our protocol does not necessarily reflect the clinical practice in other parts of the world. Fifthly, we did not include in this pilot study, an aged matched control group for evaluating changes in balance related to basal ganglia dysfunction, so we cannot conclusively ascribe our findings to basal ganglia malfunction in PD.

Finally, we want to underline that postural control measured by dynamic posturography might give more information about mechanisms of postural instability in PD than static posturography. Performing the FRT might not be as good as a test measured by dynamic posturography.

## Conclusions

This pilot study shows that a tailored balance training, in association with the sensory-motor orthotic, appears to be safe and feasible and is able to positively impact on mobility, balance, gait and quality of life. This preliminary study provides promising data on the feasibility and safety of our protocol, thus supporting the development of a large scale Randomized Control Trial. Future studies are certainly needed and will expand our knowledge on the mechanisms of action of SM orthotic, on the time needed to achieve a meaningful improvement and its long-term duration.

## Abbreviations

6mWT: Six minutes walking test
ANOVA: Analysis of Variance
BBS: Berg Balance Scale
CoP: Center of Pressure
DSM: Diagnostic and Statistical Manual of Mental Disorders
EC: Eyes closed
EO: Eyes open
FRT: Functional Reaching Test
H&Y: Hoehn and Yahr
KNGF: Koninklijk Nederlands Genootschap voor Fysiotherapie
MMSE: Mini Mental State Examination
PD: Parkinson’s disease
PDQ-39: Parkinson’s Disease Questionnaire
RM: Repeated Measures
SD: Standard Deviation
SM: Sensory motor
TUG: Time Up and Go
UPDRS: Unified Parkinson’s Disease Rating Scale
